# A multiplanar humeral head osteotomy results in significantly improved bone compression strength compared to a standard humeral head osteotomy in stemless total shoulder arthroplasty

**DOI:** 10.1016/j.jseint.2026.101631

**Published:** 2026-01-24

**Authors:** Andrew J. Frear, Victoria R. Wong, Meagan J. Makarczyk, Michael F. Shannon, Kenneth L. Urish, Matthew D. Budge

**Affiliations:** a University of Pittsburgh, School of Medicine, Pittsburgh, PA, USA; b University of Pittsburgh, Department of Orthopaedic Surgery, Pittsburgh, PA, USA; c Kaiser Permanente Northwest, Department of Orthopaedic Surgery, Salem, OR, USA

**Keywords:** Total shoulder arthroplasty, Stemless implants, Osteotomy, Multiplanar osteotomy, Compression strength, Bone strength, Humeral cut

## Abstract

**Background:**

When performing anatomic total shoulder arthroplasty, modular stemmed humeral implants are the most frequently used. However, there has been renewed interest in the use of stemless humeral components to preserve bone stock and better reproduce native anatomy. The use of stemless implants in patients with poor metaphyseal bone quality is controversial. Recently, a technique was developed using a multiplanar osteotomy (MPO) of the humeral head, which preserves the subchondral bone and potentially improves stemless implant fixation. The effect of MPO on compressive bone strength has not yet been studied. The purpose of our study is to compare the bone compression strength between humeral heads prepared with either a standard humeral head cut or an MPO.

**Methods:**

Ten matched pairs of fresh-frozen humeral heads were used. For each pair, one humeral head underwent a standard humeral neck cut using a cutting guide, with the opposite matched head undergoing an MPO. Each humeral head subsequently underwent compression testing using a 1.3 mm steel needle at a standard speed of 1 mm/s in a grid pattern across the cut surfaces of the humeral head. Maximum force was recorded and compared with each corresponding point on the opposite matched pair.

**Results:**

Mean maximum compression force across all points was significantly higher in the MPO group (97 ± 41 N) compared to the standard cut group (52 ± 26 N). Maximum compression force across each matched pair was higher in the MPO group in all samples and was statistically significant in 8 of the 10 samples.

**Conclusion:**

This study demonstrates that use of an MPO to prepare the humeral head results in significantly increased bone compression strength compared to a standard cut, with the potential to improve stemless implant fixation.

At the present time, modular stemmed humeral implants continue to be the most commonly used prosthesis when performing anatomic total shoulder arthroplasty (aTSA). However, there remain a variety of problems encountered when using traditional stemmed components, including difficulty in recreating native anatomy, stress shielding, significant bone resection, periprosthetic fracture, and complexity of revision.^4^^,^^5^^,^^7^^,^^15^^,^^19^ In response to these concerns, there has been renewed interest among shoulder surgeons in the use of stemless total shoulder arthroplasty. While recent short- and medium-term studies of some specific stemless implant designs have shown equivalency to their stemmed counterparts,^3^^,^^8^^,^^18^ there remain significant concerns about stress shielding, component migration, and early failure.^1^^,^^13^^,^^23^

Recently, a stemless prosthesis system was developed in an attempt to maximize the strength of the bony fixation of the humeral component in the subchondral bone, as opposed to the peripheral bone, by performing a multiplanar osteotomy (MPO) of the humeral head.^2^^,^^11^ This technique uses a series of cutting jigs to remove the cartilaginous surfaces of the humeral head while preserving the subchondral bone, similar to the femoral cuts on a total knee arthroplasty. In theory, this allows the thick subchondral plate to support the humeral prosthesis and improve time-zero fixation of the implant. While initial clinical studies of this design have shown good short-term results, the ability of an MPO to improve the strength and quality of the bone for purchase of the stemless humeral implant has yet to be proven.^5^^,^^11^

The purpose of this study was to determine the compression strength of the flat osteotomy surfaces of the humeral bone in a plane perpendicular to a standard anatomic humeral neck cut, between humeral heads prepared with either a traditional humeral neck osteotomy or a MPO. Our hypothesis is that humeral heads prepared with a MPO would have significantly higher compression strength compared to a traditional humeral neck osteotomy.

## Materials and methods

**Figure 1:**
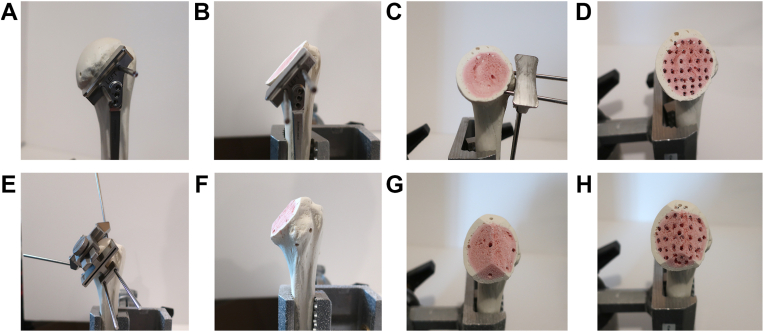
Composite picture demonstrating the differences between the standard humeral head cut (**A-D**) and MPO (**E-H**). *MPO*, multiplanar osteotomy.

Ten matched pairs of fresh-frozen proximal humeri (n = 10) were acquired from the Anatomy Gifts Registry (Hanover, MD) with oversight from the institution's Office for Oversight of Anatomic Specimens. All humeri were fully stripped of soft tissue and were inspected for any obvious signs of injury or previous surgery. Specimen were kept frozen and then thawed to room temperature for testing. For each matched pair, one sample underwent a humeral head osteotomy using a cutting jig at 135° of humeral neck angulation and humeral retroversion as determined by a fellowship trained orthopedic shoulder and elbow surgeon. The contralateral shoulder underwent a MPO using a series of cutting jigs consistent with the manufacturer's recommendations (Fig. 1).

### Compression testing

**Figure 2:**
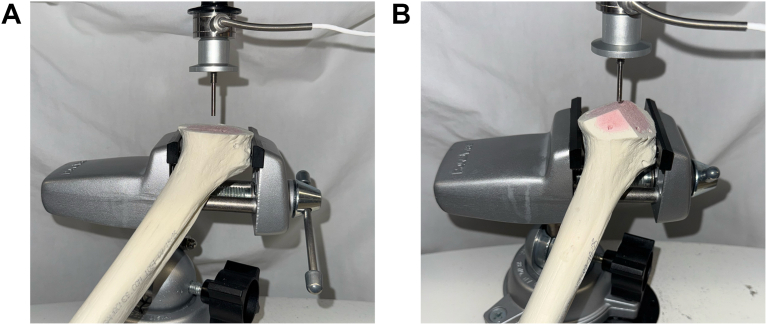
Example of compression testing set up of the standard humeral head cut and multiplanar osteotomy.

**Figure 3:**
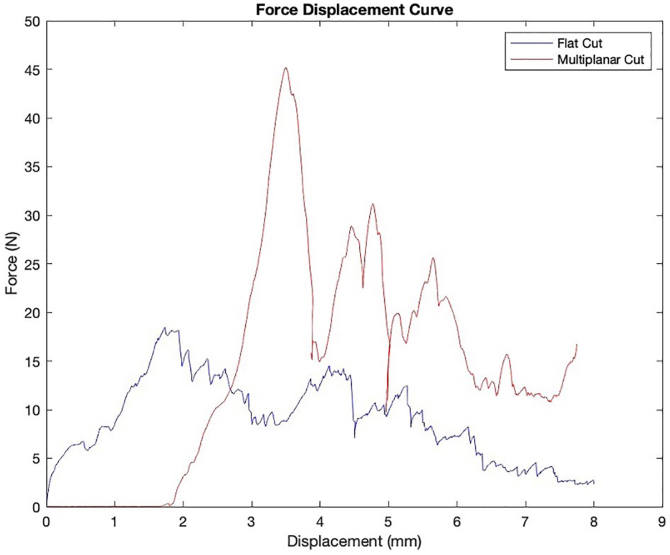
Force–displacement curve obtained during compression testing of the standard humeral head cut (in *blue*) and multiplanar osteotomy (in *red*).

Compression testing was performed using a protocol previously established by Zumstein et al.^25^ Each humeral head was divided into a standardized grid with a varying number of indentation points depending on the size of the bone surface. The number of points on each humeral head pair was the same. The distance between each point ranged between 7 and 10 mm. Each sample was fixed in a static mechanical holder for consistent pressure testing. Compression testing was performed with a 1.3 mm diameter steel needle, with a vertical speed of 1 mm/s and a standardized hole depth of 8 mm. Force in Newtons (N) was continuously obtained during testing for each indentation point (Fig. 2). Maximum force for each point was recorded and registered on a grid. Force displacement curves were generated to measure compression strength (Fig. 3).

### Statistical analysis

Patient demographics were summarized with descriptive statistics. Two-sided paired *t*-tests were used to compare each corresponding pair of points on matched humeral heads. Analyses were first conducted on all points across all patients, then separately for each individual patient. Statistical significance was defined as *P* < .05. All statistical analyses were performed on GraphPad Prism (version 10.4.0; GraphPad, San Diego, CA, USA).

## Results

### Patient demographics

The average age of the 10 donor patients included in this study was 69.3 ± 2.15 years. The patients were equally divided by sex with 5 females and 5 males in each cohort.

### Compression testing results

**Figure 4:**
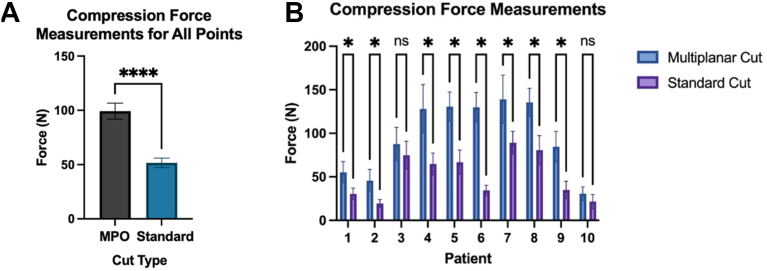
(**A**) Results of compression force testing across all points. (**B**) Results of compression force testing by individual patient. ∗ Denotes statistical significance, *P* < .05. NS denotes no statistical significance. *N*, Newtons.

The mean maximum compression force for the MPO group was 97 ± 41 N, whereas the mean maximum compression force in the standard cut group was 52 ± 26 N. This difference was statistically significant (*P* = .0001) (Fig. 4*A*). Each matched pair was similarly analyzed and individually compared. The mean maximum compression force for each individual pair is listed in Table I. For all matched pairs, the mean maximum compression forces of the MPO humeral head cut were greater than that of the standard cut group. This difference was statistically significant in 8 of the 10 patients (Table I, Fig. 4*B*).

**Table I:** Mean compression forces of Multiplanar Osteotomy and Standard Humeral Head Cut by patient.

Patient no.	MPO mean force (N)	Standard cut mean force (N)	*P* value
1	55.31	30.39	.001∗
2	45.61	19.50	.0005∗
3	87.62	74.76	.342
4	127.9	64.76	.0001∗
5	130.6	66.76	<.0001∗
6	129.7	34.36	<.0001∗
7	139.0	89.11	.0034∗
8	135.5	80.51	<.0001∗
9	84.52	34.93	.0001∗
10	30.57	21.57	.1111

*MPO*, multiplanar osteotomy.

∗ Denotes significance, *P* < .05.

## Discussion

This study compared the mechanical compression strength of proximal humeral bone between a standard humeral head osteotomy and a novel subchondral-bone preserving MPO in the setting of stemless total shoulder arthroplasty. Our results indicate that the average compressive strength of the remaining proximal humeral bone in the MPO group was significantly higher compared to that of the standard osteotomy group, both across all specimens and within matched pairs. This finding has important implications for initial fixation of stemless aTSA implants, as prior studies have shown that the relative strength and bone mineral density of the proximal humerus can affect the time-zero fixation of stemless implants.^6^^,^^9^^,^^21^ Specifically, implants which are placed in low strength bone are at risk for fibrous ingrowth with the potential for early loosening and failure.^6^^,^^10^^,^^21^

To achieve stable initial fixation, humeral implants should gain purchase either peripherally in the denser pericortical bone of the proximal humerus or in the subchondral plate.^20^^,^^21^ Saitoh et al^22^ in their seminal paper showed that the cancellous bone of the humeral neck, where the majority of stemless implant achieve fixation, has only one-third the mechanical strength of the humeral head. Similarly, Zumstein et al^25^ showed that the best bone quality of the humeral head was distributed in either a monocentric or bicentric pattern, which is in general preserved with techniques humeral preparation techniques such as an MPO or resurfacing. The majority of stemless aTSA implants currently on the market do not preserve the subchondral plate and use a standard humeral head cut, which attempts to maximize fixation in the remaining peripheral metaphyseal bone using fins, pegs, or other techniques. Using an MPO or resfurfacing technique for preparation of the humeral head is an alternative strategy that preserves both the strong peripheral metaphyseal bone as well as the subchondral plate, which can be used in fixation of stemless humeral implants. The cuts of the MPO technique differ from traditional resurfacing (Copeland, OVO) in that the angular planes of the cuts leave ridges of subchondral bone to both support the undersurface of the implant to prevent subsidence as well as to provide subchondral bone to anchor the pegs (Fig. 1). However, as with all systems that do not perform a traditional humeral head cut, there can be significant downsides, as the bulk of the remaining humeral head can impede the surgeon's ability to access the glenoid. This difficulty has been noted in other similar humeral resurfacing techniques where limited removal of humeral bone inhibits glenoid access.^17^

Using humeral bone conservation techniques such as resfurfacing or a MPO for proximal humeral preparation may decrease the need for surgeons to rely upon an intraoperative assessment of humeral bone quality. Currently when using a standard humeral head cut for preparation of the proximal humerus, surgeons rely on performing an intraoperative “thumb test” to determine if the remaining cancellous bone of the proximal humerus has adequate strength to provide stable time-zero implant fixation.^8^ This test is highly subjective and varies significantly from one surgeon to the next.^14^^,^^24^ If the humeral bone is deemed inadequate to support stemless fixation, then a stemmed implant is frequently employed requiring additional implants to be available. Although more recent radiologic based determinations of proximal bone density have shown promise in predicting adequate proximal humeral bone strength,^16^ the use of humeral bone conservation techniques potentially removes the need to rely on a subjective judgment of bone quality, as the compressive strength of the bone should be much improved by preserving the subchondral plate.

The results of our study indicate that the use of an MPO when performing a stemless aTSA provides significantly improved proximal humeral bone compressive strength, in theory leading to improved time-zero fixation. Average bone compression strength was almost double in the MPO cohort compared to the standard humeral head cut group and in one individual matched pair the MPO compression strength was triple. Recent short term clinical data on implants using this technique showed no loosening or failures of the humeral component at five years, even in elderly individuals who would be at higher risk for having low bone mineral density.^12^

Limitations of this study include a lack of measurement of bone mineral density prior to mechanical testing and using an osteotomy technique that is unique to one specific implant. While measuring bone mineral density may have been useful from a scientific standpoint, it is a proxy for determining the actual physical strength of the humeral bone. We feel this was measured appropriately with mechanical testing and has more clinical impact on initial implant fixation. Similarly, the use of matched pairs of shoulders from the same donor should decrease the consequence of varied bone mineral density between the 2 osteotomy types and mitigates the need for bone mineral density testing. In terms of the MPO being a novel technique specific to one implant, we believe that this study shows the utility of considering different methods of proximal humeral preparation for shoulder implants. This could potentially spark interest in other novel methods to preserve humeral bone in the future. Strengths of this study include the use of matched proximal humeral pairs to decrease variability in anatomy and bone mineral density among the shoulders, and the use of a well-described uniform testing protocol for the entirety of the proximal humeral bone.

## Conclusion

In the setting of stemless total shoulder arthroplasty, the use of a MPO to prepare the proximal humerus results in a significant increase in bone compression strength compared to a standard humeral neck osteotomy.

## Disclaimers:

Funding: Supported by the National Institute of Arthritis and Musculoskeletal and Skin Diseases, Project Number 5R01AR082167-03.

Conflicts of interest: Dr Matthew D. Budge is a consultant for Catalyst Orthoscience, and has stock or stock options for Catalyst Orthoscience. Dr Kenneth Urish is supported by NIH NIAMS (R01 AR082167) which supported this research study. The other authors, their immediate families, and any research foundation with which they are affiliated have not received any financial payments or other benefits from any commercial entity related to the subject of this article.
